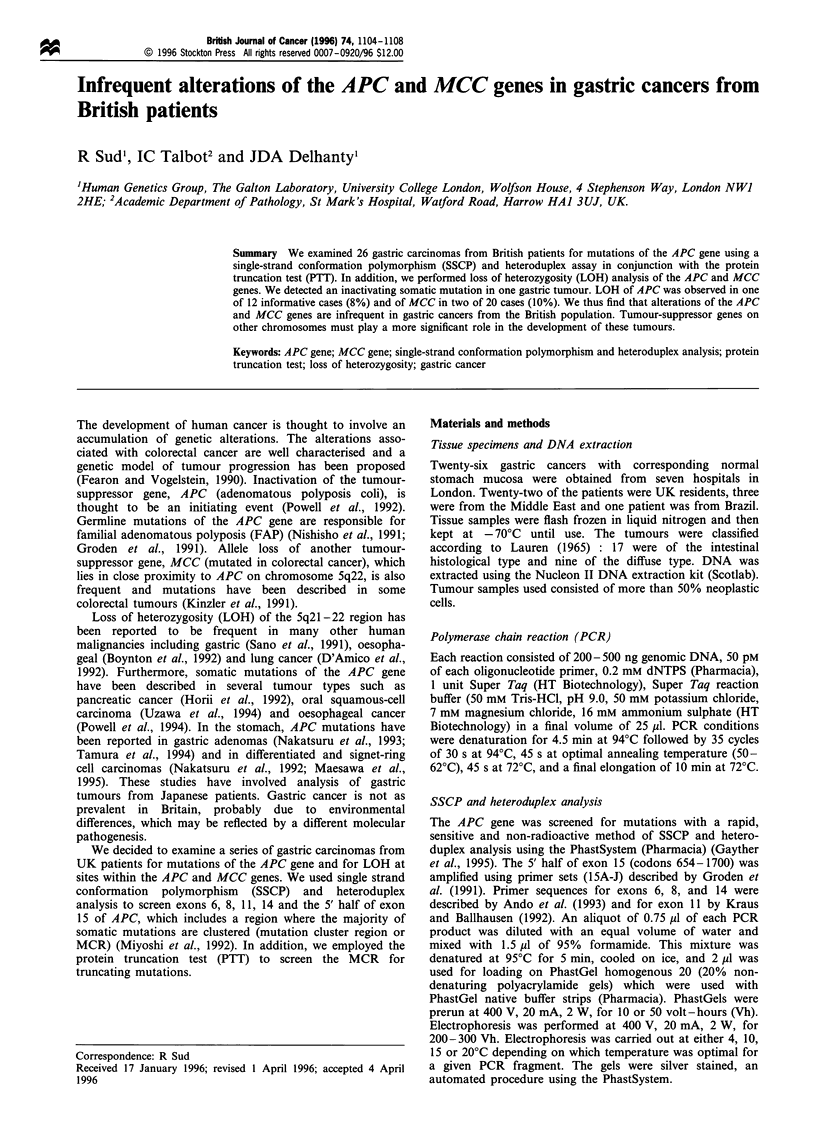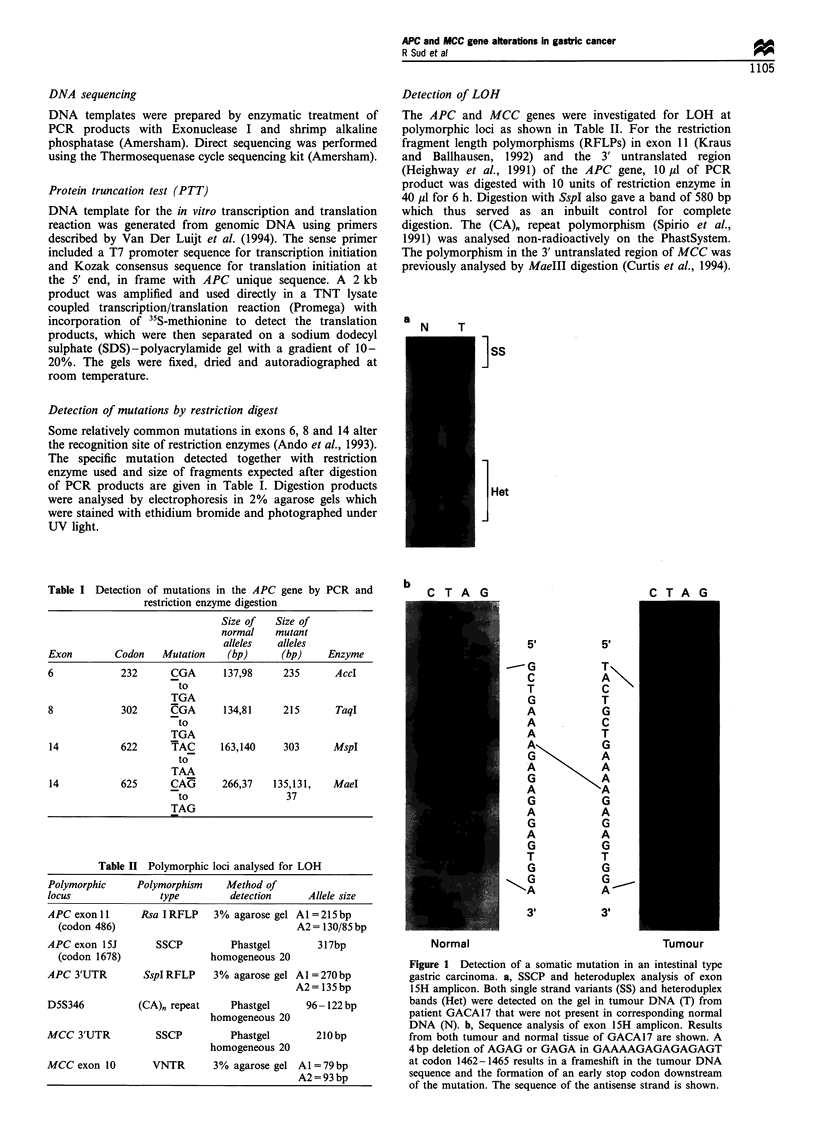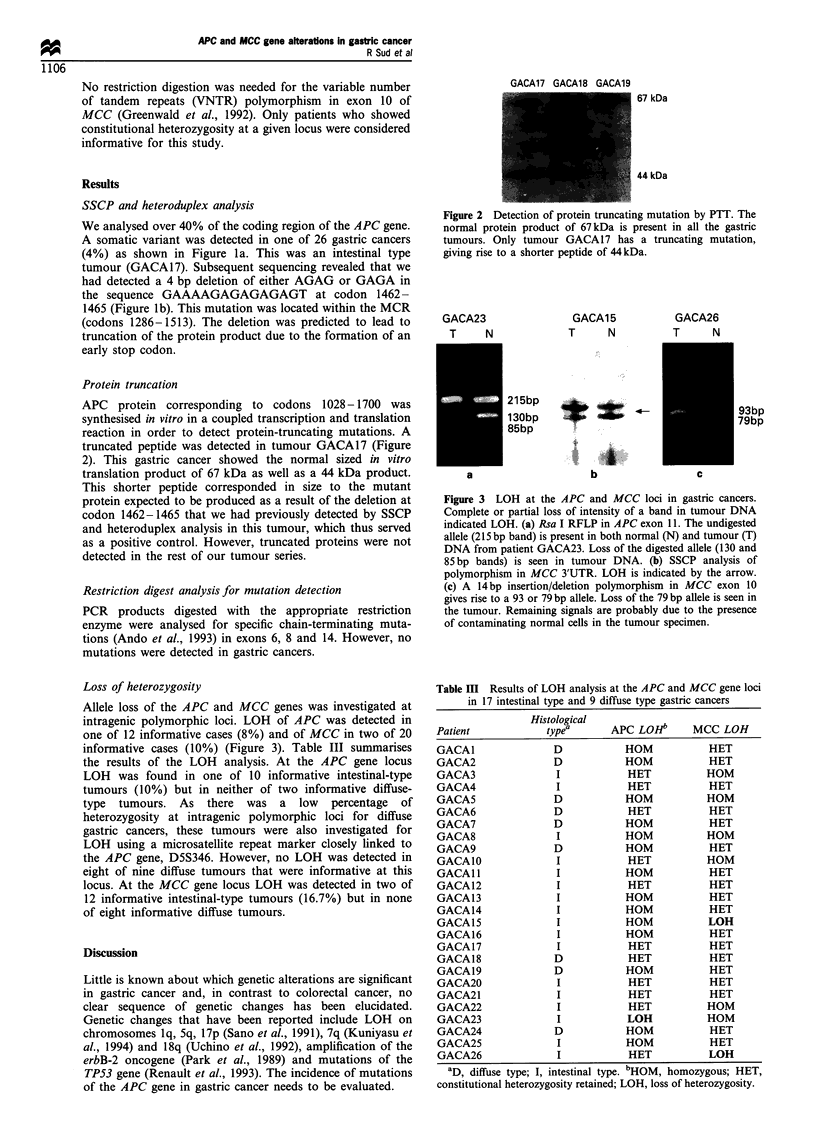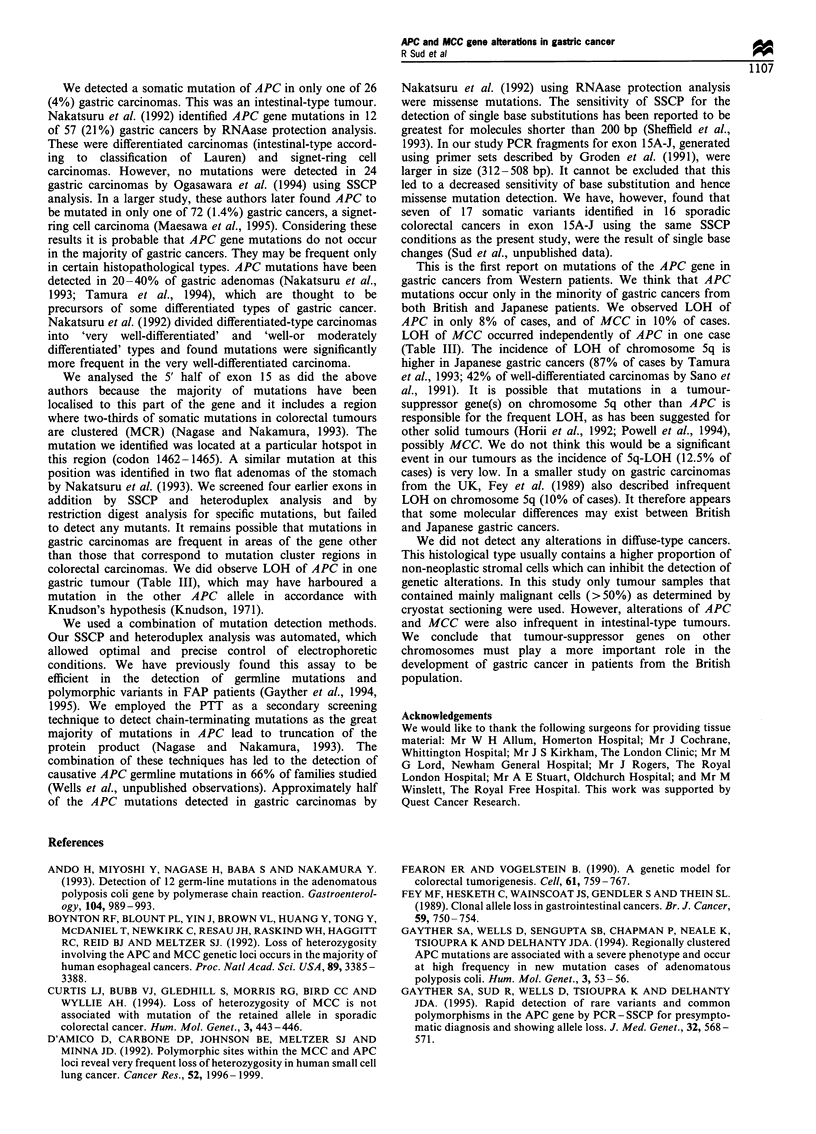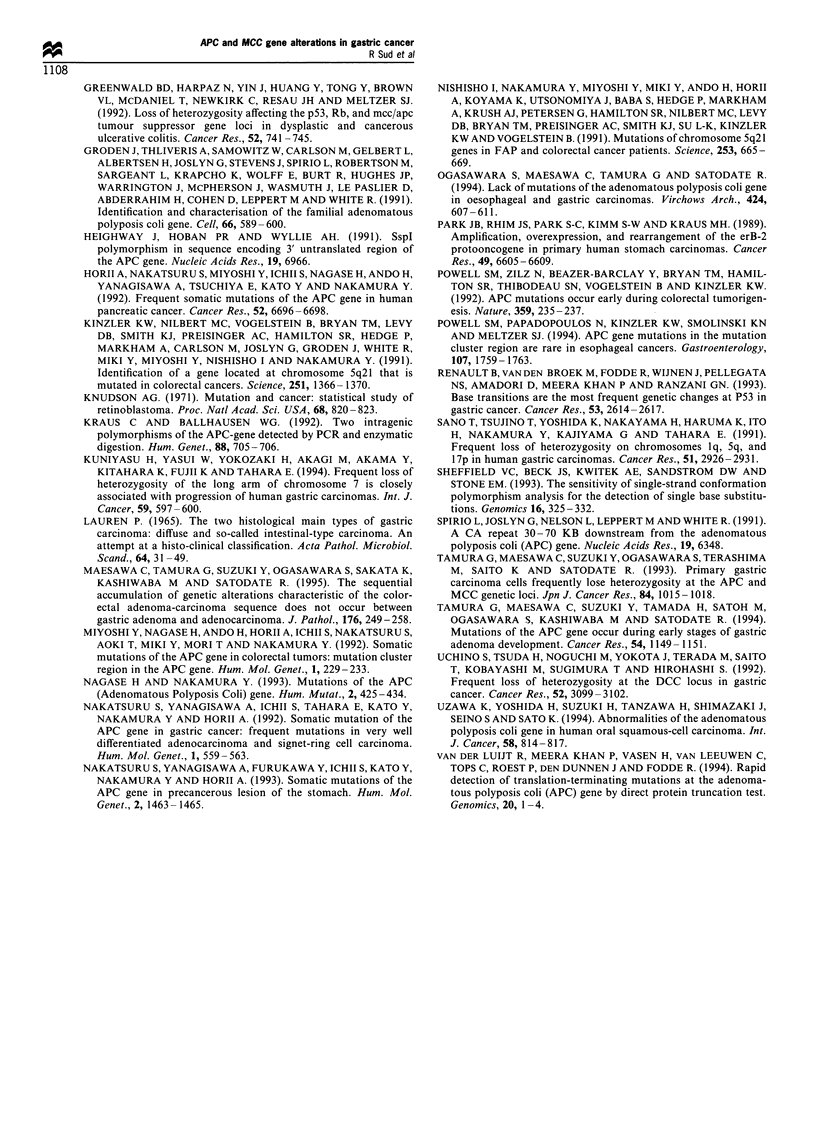# Infrequent alterations of the APC and MCC genes in gastric cancers from British patients.

**DOI:** 10.1038/bjc.1996.497

**Published:** 1996-10

**Authors:** R. Sud, I. C. Talbot, J. D. Delhanty

**Affiliations:** Human Genetics Group, Galton Laboratory, University College London, UK.

## Abstract

**Images:**


British Journal of Cancer (1996) 74, 1104-1108
© 1996 Stockton Press All rights reserved 0007-0920/96 $12.00

### Infrequent alterations of the *APC* and *MCC* genes in gastric cancers from British patients

R Sud[1], IC Talbot[2] and JDA Delhanty[1]

[1]*Human Genetics Group, The Galton Laboratory, University College London, Wolfson House, 4 Stephenson Way, London NW1 2HE;* [2]*Academic Department of Pathology, St Mark's Hospital, Watford Road, Harrow HA1 3UJ, UK.*

**Summary** We examined 26 gastric carcinomas from British patients for mutations of the *APC* gene using a single-strand conformation polymorphism (SSCP) and heteroduplex assay in conjunction with the protein truncation test (PTT). In addition, we performed loss of heterozygosity (LOH) analysis of the *APC* and *MCC* genes. We detected an inactivating somatic mutation in one gastric tumour. LOH of *APC* was observed in one of 12 informative cases (8%) and of *MCC* in two of 20 cases (10%). We thus find that alterations of the *APC* and *MCC* genes are infrequent in gastric cancers from the British population. Tumour-suppressor genes on other chromosomes must play a more significant role in the development of these tumours.

**Keywords:** *APC* gene; *MCC* gene; single-strand conformation polymorphism and heteroduplex analysis; protein truncation test; loss of heterozygosity; gastric cancer

The development of human cancer is thought to involve an accumulation of genetic alterations. The alterations associated with colorectal cancer are well characterised and a genetic model of tumour progression has been proposed (Fearon and Vogelstein, 1990). Inactivation of the tumour-suppressor gene, *APC* (adenomatous polyposis coli), is thought to be an initiating event (Powell *et al.*, 1992). Germline mutations of the *APC* gene are responsible for familial adenomatous polyposis (FAP) (Nishisho *et al.*, 1991; Groden *et al.*, 1991). Allele loss of another tumour-suppressor gene, *MCC* (mutated in colorectal cancer), which lies in close proximity to *APC* on chromosome 5q22, is also frequent and mutations have been described in some colorectal tumours (Kinzler *et al.*, 1991).

Loss of heterozygosity (LOH) of the 5q21-22 region has been reported to be frequent in many other human malignancies including gastric (Sano *et al.*, 1991), oesophageal (Boynton *et al.*, 1992) and lung cancer (D'Amico *et al.*, 1992). Furthermore, somatic mutations of the *APC* gene have been described in several tumour types such as pancreatic cancer (Horii *et al.*, 1992), oral squamous-cell carcinoma (Uzawa *et al.*, 1994) and oesophageal cancer (Powell *et al.*, 1994). In the stomach, *APC* mutations have been reported in gastric adenomas (Nakatsuru *et al.*, 1993; Tamura *et al.*, 1994) and in differentiated and signet-ring cell carcinomas (Nakatsuru *et al.*, 1992; Maesawa *et al.*, 1995). These studies have involved analysis of gastric tumours from Japanese patients. Gastric cancer is not as prevalent in Britain, probably due to environmental differences, which may be reflected by a different molecular pathogenesis.

We decided to examine a series of gastric carcinomas from UK patients for mutations of the *APC* gene and for LOH at sites within the *APC* and *MCC* genes. We used single strand conformation polymorphism (SSCP) and heteroduplex analysis to screen exons 6, 8, 11, 14 and the 5′ half of exon 15 of *APC*, which includes a region where the majority of somatic mutations are clustered (mutation cluster region or MCR) (Miyoshi *et al.*, 1992). In addition, we employed the protein truncation test (PTT) to screen the MCR for truncating mutations.

Correspondence: R Sud
Received 17 January 1996; revised 1 April 1996; accepted 4 April 1996

### Materials and methods

### *Tissue specimens and DNA extraction*

Twenty-six gastric cancers with corresponding normal stomach mucosa were obtained from seven hospitals in London. Twenty-two of the patients were UK residents, three were from the Middle East and one patient was from Brazil. Tissue samples were flash frozen in liquid nitrogen and then kept at −70°C until use. The tumours were classified according to Lauren (1965) : 17 were of the intestinal histological type and nine of the diffuse type. DNA was extracted using the Nucleon II DNA extraction kit (Scotlab). Tumour samples used consisted of more than 50% neoplastic cells.

### *Polymerase chain reaction (PCR)*

Each reaction consisted of 200-500 ng genomic DNA, 50 pM of each oligonucleotide primer, 0.2 mM dNTPS (Pharmacia), 1 unit Super *Taq* (HT Biotechnology), Super *Taq* reaction buffer (50 mM Tris-HCl, pH 9.0, 50 mM potassium chloride, 7 mM magnesium chloride, 16 mM ammonium sulphate (HT Biotechnology) in a final volume of 25 μl. PCR conditions were denaturation for 4.5 min at 94°C followed by 35 cycles of 30 s at 94°C, 45 s at optimal annealing temperature (50-62°C), 45 s at 72°C, and a final elongation of 10 min at 72°C.

### *SSCP and heteroduplex analysis*

The *APC* gene was screened for mutations with a rapid, sensitive and non-radioactive method of SSCP and heteroduplex analysis using the PhastSystem (Pharmacia) (Gayther *et al.*, 1995). The 5′ half of exon 15 (codons 654-1700) was amplified using primer sets (15A-J) described by Groden *et al.* (1991). Primer sequences for exons 6, 8, and 14 were described by Ando *et al.* (1993) and for exon 11 by Kraus and Ballhausen (1992). An aliquot of 0.75 μl of each PCR product was diluted with an equal volume of water and mixed with 1.5 μl of 95% formamide. This mixture was denatured at 95°C for 5 min, cooled on ice, and 2 μl was used for loading on PhastGel homogenous 20 (20% non-denaturing polyacrylamide gels) which were used with PhastGel native buffer strips (Pharmacia). PhastGels were prerun at 400 V, 20 mA, 2 W, for 10 or 50 volt-hours (Vh). Electrophoresis was performed at 400 V, 20 mA, 2 W, for 200-300 Vh. Electrophoresis was carried out at either 4, 10, 15 or 20°C depending on which temperature was optimal for a given PCR fragment. The gels were silver stained, an automated procedure using the PhastSystem.

*DNA sequencing*

DNA templates were prepared by enzymatic treatment of PCR products with Exonuclease I and shrimp alkaline phosphatase (Amersham). Direct sequencing was performed using the Thermosequenase cycle sequencing kit (Amersham).

*Protein truncation test (PTT)*

DNA template for the *in vitro* transcription and translation reaction was generated from genomic DNA using primers described by Van Der Luijt *et al.* (1994). The sense primer included a T7 promoter sequence for transcription initiation and Kozak consensus sequence for translation initiation at the 5′ end, in frame with *APC* unique sequence. A 2 kb product was amplified and used directly in a TNT lysate coupled transcription/translation reaction (Promega) with incorporation of $^{35}$S-methionine to detect the translation products, which were then separated on a sodium dodecyl sulphate (SDS)–polyacrylamide gel with a gradient of 10–20%. The gels were fixed, dried and autoradiographed at room temperature.

*Detection of mutations by restriction digest*

Some relatively common mutations in exons 6, 8 and 14 alter the recognition site of restriction enzymes (Ando *et al.*, 1993). The specific mutation detected together with restriction enzyme used and size of fragments expected after digestion of PCR products are given in Table I. Digestion products were analysed by electrophoresis in 2% agarose gels which were stained with ethidium bromide and photographed under UV light.

**Table I** Detection of mutations in the *APC* gene by PCR and restriction enzyme digestion

| *Exon* | *Codon* | *Mutation* | *Size of normal alleles (bp)* | *Size of mutant alleles (bp)* | *Enzyme* |
|---|---|---|---|---|---|
| 6 | 232 | CGA to TGA | 137,98 | 235 | *Acc*I |
| 8 | 302 | CGA to TGA | 134,81 | 215 | *Taq*I |
| 14 | 622 | TAC to TAA | 163,140 | 303 | *Msp*I |
| 14 | 625 | CAG to TAG | 266,37 | 135,131, 37 | *Mae*I |

**Table II** Polymorphic loci analysed for LOH

| *Polymorphic locus* | *Polymorphism type* | *Method of detection* | *Allele size* |
|---|---|---|---|
| *APC* exon 11 (codon 486) | *Rsa* I RFLP | 3% agarose gel | A1 = 215 bp A2 = 130/85 bp |
| *APC* exon 15J (codon 1678) | SSCP | Phastgel homogeneous 20 | 317bp |
| *APC* 3′UTR | *Ssp*I RFLP | 3% agarose gel | A1 = 270 bp A2 = 135 bp |
| D5S346 | $(CA)_n$ repeat | Phastgel homogeneous 20 | 96–122 bp |
| *MCC* 3′UTR | SSCP | Phastgel homogeneous 20 | 210 bp |
| *MCC* exon 10 | VNTR | 3% agarose gel | A1 = 79 bp A2 = 93 bp |

*Detection of LOH*

The *APC* and *MCC* genes were investigated for LOH at polymorphic loci as shown in Table II. For the restriction fragment length polymorphisms (RFLPs) in exon 11 (Kraus and Ballhausen, 1992) and the 3′ untranslated region (Heighway *et al.*, 1991) of the *APC* gene, 10 μl of PCR product was digested with 10 units of restriction enzyme in 40 μl for 6 h. Digestion with *Ssp*I also gave a band of 580 bp which thus served as an inbuilt control for complete digestion. The $(CA)_n$ repeat polymorphism (Spirio *et al.*, 1991) was analysed non-radioactively on the PhastSystem. The polymorphism in the 3′ untranslated region of *MCC* was previously analysed by *Mae*III digestion (Curtis *et al.*, 1994).

**Figure 1** Detection of a somatic mutation in an intestinal type gastric carcinoma. **a**, SSCP and heteroduplex analysis of exon 15H amplicon. Both single strand variants (SS) and heteroduplex bands (Het) were detected on the gel in tumour DNA (T) from patient GACA17 that were not present in corresponding normal DNA (N). **b**, Sequence analysis of exon 15H amplicon. Results from both tumour and normal tissue of GACA17 are shown. A 4 bp deletion of AGAG or GAGA in GAAAAGAGAGAGAGT at codon 1462–1465 results in a frameshift in the tumour DNA sequence and the formation of an early stop codon downstream of the mutation. The sequence of the antisense strand is shown.

No restriction digestion was needed for the variable number of tandem repeats (VNTR) polymorphism in exon 10 of *MCC* (Greenwald *et al.*, 1992). Only patients who showed constitutional heterozygosity at a given locus were considered informative for this study.

### Results

### *SSCP and heteroduplex analysis*

We analysed over 40% of the coding region of the *APC* gene. A somatic variant was detected in one of 26 gastric cancers (4%) as shown in Figure 1a. This was an intestinal type tumour (GACA17). Subsequent sequencing revealed that we had detected a 4 bp deletion of either AGAG or GAGA in the sequence GAAAAGAGAGAGAGT at codon 1462–1465 (Figure 1b). This mutation was located within the MCR (codons 1286–1513). The deletion was predicted to lead to truncation of the protein product due to the formation of an early stop codon.

### *Protein truncation*

APC protein corresponding to codons 1028–1700 was synthesised *in vitro* in a coupled transcription and translation reaction in order to detect protein-truncating mutations. A truncated peptide was detected in tumour GACA17 (Figure 2). This gastric cancer showed the normal sized *in vitro* translation product of 67 kDa as well as a 44 kDa product. This shorter peptide corresponded in size to the mutant protein expected to be produced as a result of the deletion at codon 1462–1465 that we had previously detected by SSCP and heteroduplex analysis in this tumour, which thus served as a positive control. However, truncated proteins were not detected in the rest of our tumour series.

### *Restriction digest analysis for mutation detection*

PCR products digested with the appropriate restriction enzyme were analysed for specific chain-terminating mutations (Ando *et al.*, 1993) in exons 6, 8 and 14. However, no mutations were detected in gastric cancers.

### *Loss of heterozygosity*

Allele loss of the *APC* and *MCC* genes was investigated at intragenic polymorphic loci. LOH of *APC* was detected in one of 12 informative cases (8%) and of *MCC* in two of 20 informative cases (10%) (Figure 3). Table III summarises the results of the LOH analysis. At the *APC* gene locus LOH was found in one of 10 informative intestinal-type tumours (10%) but in neither of two informative diffuse-type tumours. As there was a low percentage of heterozygosity at intragenic polymorphic loci for diffuse gastric cancers, these tumours were also investigated for LOH using a microsatellite repeat marker closely linked to the *APC* gene, D5S346. However, no LOH was detected in eight of nine diffuse tumours that were informative at this locus. At the *MCC* gene locus LOH was detected in two of 12 informative intestinal-type tumours (16.7%) but in none of eight informative diffuse tumours.

### Discussion

Little is known about which genetic alterations are significant in gastric cancer and, in contrast to colorectal cancer, no clear sequence of genetic changes has been elucidated. Genetic changes that have been reported include LOH on chromosomes 1q, 5q, 17p (Sano *et al.*, 1991), 7q (Kuniyasu *et al.*, 1994) and 18q (Uchino *et al.*, 1992), amplification of the *erb*B-2 oncogene (Park *et al.*, 1989) and mutations of the *TP53* gene (Renault *et al.*, 1993). The incidence of mutations of the *APC* gene in gastric cancer needs to be evaluated.

**Figure 2** Detection of protein truncating mutation by PTT. The normal protein product of 67 kDa is present in all the gastric tumours. Only tumour GACA17 has a truncating mutation, giving rise to a shorter peptide of 44 kDa.

**Figure 3** LOH at the *APC* and *MCC* loci in gastric cancers. Complete or partial loss of intensity of a band in tumour DNA indicated LOH. (**a**) *Rsa* I RFLP in *APC* exon 11. The undigested allele (215 bp band) is present in both normal (N) and tumour (T) DNA from patient GACA23. Loss of the digested allele (130 and 85 bp bands) is seen in tumour DNA. (**b**) SSCP analysis of polymorphism in *MCC* 3'UTR. LOH is indicated by the arrow. (**c**) A 14 bp insertion/deletion polymorphism in *MCC* exon 10 gives rise to a 93 or 79 bp allele. Loss of the 79 bp allele is seen in the tumour. Remaining signals are probably due to the presence of contaminating normal cells in the tumour specimen.

**Table III** Results of LOH analysis at the *APC* and *MCC* gene loci in 17 intestinal type and 9 diffuse type gastric cancers

| *Patient* | *Histological type*[a] | APC *LOH*[b] | MCC *LOH* |
|---|---|---|---|
| GACA1 | D | HOM | HET |
| GACA2 | D | HOM | HET |
| GACA3 | I | HET | HOM |
| GACA4 | I | HET | HET |
| GACA5 | D | HOM | HOM |
| GACA6 | D | HET | HET |
| GACA7 | D | HOM | HET |
| GACA8 | I | HOM | HOM |
| GACA9 | D | HOM | HET |
| GACA10 | I | HET | HOM |
| GACA11 | I | HOM | HET |
| GACA12 | I | HET | HET |
| GACA13 | I | HOM | HET |
| GACA14 | I | HOM | HET |
| GACA15 | I | HOM | **LOH** |
| GACA16 | I | HOM | HET |
| GACA17 | I | HET | HET |
| GACA18 | D | HET | HET |
| GACA19 | D | HOM | HET |
| GACA20 | I | HET | HET |
| GACA21 | I | HET | HET |
| GACA22 | I | HET | HOM |
| GACA23 | I | **LOH** | HOM |
| GACA24 | D | HOM | HET |
| GACA25 | I | HOM | HET |
| GACA26 | I | HET | **LOH** |

[a]D, diffuse type; I, intestinal type. [b]HOM, homozygous; HET, constitutional heterozygosity retained; LOH, loss of heterozygosity.

We detected a somatic mutation of *APC* in only one of 26 (4%) gastric carcinomas. This was an intestinal-type tumour. Nakatsuru *et al.* (1992) identified *APC* gene mutations in 12 of 57 (21%) gastric cancers by RNAase protection analysis. These were differentiated carcinomas (intestinal-type according to classification of Lauren) and signet-ring cell carcinomas. However, no mutations were detected in 24 gastric carcinomas by Ogasawara *et al.* (1994) using SSCP analysis. In a larger study, these authors later found *APC* to be mutated in only one of 72 (1.4%) gastric cancers, a signet-ring cell carcinoma (Maesawa *et al.*, 1995). Considering these results it is probable that *APC* gene mutations do not occur in the majority of gastric cancers. They may be frequent only in certain histopathological types. *APC* mutations have been detected in 20–40% of gastric adenomas (Nakatsuru *et al.*, 1993; Tamura *et al.*, 1994), which are thought to be precursors of some differentiated types of gastric cancer. Nakatsuru *et al.* (1992) divided differentiated-type carcinomas into 'very well-differentiated' and 'well-or moderately differentiated' types and found mutations were significantly more frequent in the very well-differentiated carcinoma.

We analysed the 5′ half of exon 15 as did the above authors because the majority of mutations have been localised to this part of the gene and it includes a region where two-thirds of somatic mutations in colorectal tumours are clustered (MCR) (Nagase and Nakamura, 1993). The mutation we identified was located at a particular hotspot in this region (codon 1462–1465). A similar mutation at this position was identified in two flat adenomas of the stomach by Nakatsuru *et al.* (1993). We screened four earlier exons in addition by SSCP and heteroduplex analysis and by restriction digest analysis for specific mutations, but failed to detect any mutants. It remains possible that mutations in gastric carcinomas are frequent in areas of the gene other than those that correspond to mutation cluster regions in colorectal carcinomas. We did observe LOH of *APC* in one gastric tumour (Table III), which may have harboured a mutation in the other *APC* allele in accordance with Knudson's hypothesis (Knudson, 1971).

We used a combination of mutation detection methods. Our SSCP and heteroduplex analysis was automated, which allowed optimal and precise control of electrophoretic conditions. We have previously found this assay to be efficient in the detection of germline mutations and polymorphic variants in FAP patients (Gayther *et al.*, 1994, 1995). We employed the PTT as a secondary screening technique to detect chain-terminating mutations as the great majority of mutations in *APC* lead to truncation of the protein product (Nagase and Nakamura, 1993). The combination of these techniques has led to the detection of causative *APC* germline mutations in 66% of families studied (Wells *et al.*, unpublished observations). Approximately half of the *APC* mutations detected in gastric carcinomas by Nakatsuru *et al.* (1992) using RNAase protection analysis were missense mutations. The sensitivity of SSCP for the detection of single base substitutions has been reported to be greatest for molecules shorter than 200 bp (Sheffield *et al.*, 1993). In our study PCR fragments for exon 15A-J, generated using primer sets described by Groden *et al.* (1991), were larger in size (312–508 bp). It cannot be excluded that this led to a decreased sensitivity of base substitution and hence missense mutation detection. We have, however, found that seven of 17 somatic variants identified in 16 sporadic colorectal cancers in exon 15A-J using the same SSCP conditions as the present study, were the result of single base changes (Sud *et al.*, unpublished data).

This is the first report on mutations of the *APC* gene in gastric cancers from Western patients. We think that *APC* mutations occur only in the minority of gastric cancers from both British and Japanese patients. We observed LOH of *APC* in only 8% of cases, and of *MCC* in 10% of cases. LOH of *MCC* occurred independently of *APC* in one case (Table III). The incidence of LOH of chromosome 5q is higher in Japanese gastric cancers (87% of cases by Tamura *et al.*, 1993; 42% of well-differentiated carcinomas by Sano *et al.*, 1991). It is possible that mutations in a tumour-suppressor gene(s) on chromosome 5q other than *APC* is responsible for the frequent LOH, as has been suggested for other solid tumours (Horii *et al.*, 1992; Powell *et al.*, 1994), possibly *MCC*. We do not think this would be a significant event in our tumours as the incidence of 5q-LOH (12.5% of cases) is very low. In a smaller study on gastric carcinomas from the UK, Fey *et al.* (1989) also described infrequent LOH on chromosome 5q (10% of cases). It therefore appears that some molecular differences may exist between British and Japanese gastric cancers.

We did not detect any alterations in diffuse-type cancers. This histological type usually contains a higher proportion of non-neoplastic stromal cells which can inhibit the detection of genetic alterations. In this study only tumour samples that contained mainly malignant cells (>50%) as determined by cryostat sectioning were used. However, alterations of *APC* and *MCC* were also infrequent in intestinal-type tumours. We conclude that tumour-suppressor genes on other chromosomes must play a more important role in the development of gastric cancer in patients from the British population.

**Acknowledgements**

We would like to thank the following surgeons for providing tissue material: Mr W H Allum, Homerton Hospital; Mr J Cochrane, Whittington Hospital; Mr J S Kirkham, The London Clinic; Mr M G Lord, Newham General Hospital; Mr J Rogers, The Royal London Hospital; Mr A E Stuart, Oldchurch Hospital; and Mr M Winslett, The Royal Free Hospital. This work was supported by Quest Cancer Research.

### References

ANDO H, MIYOSHI Y, NAGASE H, BABA S AND NAKAMURA Y. (1993). Detection of 12 germ-line mutations in the adenomatous polyposis coli gene by polymerase chain reaction. *Gastroenterology*, **104**, 989–993.

BOYNTON RF, BLOUNT PL, YIN J, BROWN VL, HUANG Y, TONG Y, McDANIEL T, NEWKIRK C, RESAU JH, RASKIND WH, HAGGITT RC, REID BJ AND MELTZER SJ. (1992). Loss of heterozygosity involving the APC and MCC genetic loci occurs in the majority of human esophageal cancers. *Proc. Natl Acad. Sci. USA*, **89**, 3385–3388.

CURTIS LJ, BUBB VJ, GLEDHILL S, MORRIS RG, BIRD CC AND WYLLIE AH. (1994). Loss of heterozygosity of MCC is not associated with mutation of the retained allele in sporadic colorectal cancer. *Hum. Mol. Genet.*, **3**, 443–446.

D'AMICO D, CARBONE DP, JOHNSON BE, MELTZER SJ AND MINNA JD. (1992). Polymorphic sites within the MCC and APC loci reveal very frequent loss of heterozygosity in human small cell lung cancer. *Cancer Res.*, **52**, 1996–1999.

FEARON ER AND VOGELSTEIN B. (1990). A genetic model for colorectal tumorigenesis. *Cell*, **61**, 759–767.

FEY MF, HESKETH C, WAINSCOAT JS, GENDLER S AND THEIN SL. (1989). Clonal allele loss in gastrointestinal cancers. *Br. J. Cancer*, **59**, 750–754.

GAYTHER SA, WELLS D, SENGUPTA SB, CHAPMAN P, NEALE K, TSIOUPRA K AND DELHANTY JDA. (1994). Regionally clustered APC mutations are associated with a severe phenotype and occur at high frequency in new mutation cases of adenomatous polyposis coli. *Hum. Mol. Genet.*, **3**, 53–56.

GAYTHER SA, SUD R, WELLS D, TSIOUPRA K AND DELHANTY JDA. (1995). Rapid detection of rare variants and common polymorphisms in the APC gene by PCR–SSCP for presymptomatic diagnosis and showing allele loss. *J. Med. Genet.*, **32**, 568–571.

GREENWALD BD, HARPAZ N, YIN J, HUANG Y, TONG Y, BROWN VL, MCDANIEL T, NEWKIRK C, RESAU JH AND MELTZER SJ. (1992). Loss of heterozygosity affecting the p53, Rb, and mcc/apc tumour suppressor gene loci in dysplastic and cancerous ulcerative colitis. *Cancer Res.*, **52,** 741–745.

GRODEN J, THLIVERIS A, SAMOWITZ W, CARLSON M, GELBERT L, ALBERTSEN H, JOSLYN G, STEVENS J, SPIRIO L, ROBERTSON M, SARGEANT L, KRAPCHO K, WOLFF E, BURT R, HUGHES JP, WARRINGTON J, MCPHERSON J, WASMUTH J, LE PASLIER D, ABDERRAHIM H, COHEN D, LEPPERT M AND WHITE R. (1991). Identification and characterisation of the familial adenomatous polyposis coli gene. *Cell*, **66,** 589–600.

HEIGHWAY J, HOBAN PR AND WYLLIE AH. (1991). SspI polymorphism in sequence encoding 3′ untranslated region of the APC gene. *Nucleic Acids Res.*, **19,** 6966.

HORII A, NAKATSURU S, MIYOSHI Y, ICHII S, NAGASE H, ANDO H, YANAGISAWA A, TSUCHIYA E, KATO Y AND NAKAMURA Y. (1992). Frequent somatic mutations of the APC gene in human pancreatic cancer. *Cancer Res.*, **52,** 6696–6698.

KINZLER KW, NILBERT MC, VOGELSTEIN B, BRYAN TM, LEVY DB, SMITH KJ, PREISINGER AC, HAMILTON SR, HEDGE P, MARKHAM A, CARLSON M, JOSLYN G, GRODEN J, WHITE R, MIKI Y, MIYOSHI Y, NISHISHO I AND NAKAMURA Y. (1991). Identification of a gene located at chromosome 5q21 that is mutated in colorectal cancers. *Science*, **251,** 1366–1370.

KNUDSON AG. (1971). Mutation and cancer: statistical study of retinoblastoma. *Proc. Natl Acad. Sci. USA*, **68,** 820–823.

KRAUS C AND BALLHAUSEN WG. (1992). Two intragenic polymorphisms of the APC-gene detected by PCR and enzymatic digestion. *Hum. Genet.*, **88,** 705–706.

KUNIYASU H, YASUI W, YOKOZAKI H, AKAGI M, AKAMA Y, KITAHARA K, FUJII K AND TAHARA E. (1994). Frequent loss of heterozygosity of the long arm of chromosome 7 is closely associated with progression of human gastric carcinomas. *Int. J. Cancer*, **59,** 597–600.

LAUREN P. (1965). The two histological main types of gastric carcinoma: diffuse and so-called intestinal-type carcinoma. An attempt at a histo-clinical classification. *Acta Pathol. Microbiol. Scand.*, **64,** 31–49.

MAESAWA C, TAMURA G, SUZUKI Y, OGASAWARA S, SAKATA K, KASHIWABA M AND SATODATE R. (1995). The sequential accumulation of genetic alterations characteristic of the colorectal adenoma-carcinoma sequence does not occur between gastric adenoma and adenocarcinoma. *J. Pathol.*, **176,** 249–258.

MIYOSHI Y, NAGASE H, ANDO H, HORII A, ICHII S, NAKATSURU S, AOKI T, MIKI Y, MORI T AND NAKAMURA Y. (1992). Somatic mutations of the APC gene in colorectal tumors: mutation cluster region in the APC gene. *Hum. Mol. Genet.*, **1,** 229–233.

NAGASE H AND NAKAMURA Y. (1993). Mutations of the APC (Adenomatous Polyposis Coli) gene. *Hum. Mutat.*, **2,** 425–434.

NAKATSURU S, YANAGISAWA A, ICHII S, TAHARA E, KATO Y, NAKAMURA Y AND HORII A. (1992). Somatic mutation of the APC gene in gastric cancer: frequent mutations in very well differentiated adenocarcinoma and signet-ring cell carcinoma. *Hum. Mol. Genet.*, **1,** 559–563.

NAKATSURU S, YANAGISAWA A, FURUKAWA Y, ICHII S, KATO Y, NAKAMURA Y AND HORII A. (1993). Somatic mutations of the APC gene in precancerous lesion of the stomach. *Hum. Mol. Genet.*, **2,** 1463–1465.

NISHISHO I, NAKAMURA Y, MIYOSHI Y, MIKI Y, ANDO H, HORII A, KOYAMA K, UTSONOMIYA J, BABA S, HEDGE P, MARKHAM A, KRUSH AJ, PETERSEN G, HAMILTON SR, NILBERT MC, LEVY DB, BRYAN TM, PREISINGER AC, SMITH KJ, SU L-K, KINZLER KW AND VOGELSTEIN B. (1991). Mutations of chromosome 5q21 genes in FAP and colorectal cancer patients. *Science*, **253,** 665–669.

OGASAWARA S, MAESAWA C, TAMURA G AND SATODATE R. (1994). Lack of mutations of the adenomatous polyposis coli gene in oesophageal and gastric carcinomas. *Virchows Arch.*, **424,** 607–611.

PARK JB, RHIM JS, PARK S-C, KIMM S-W AND KRAUS MH. (1989). Amplification, overexpression, and rearrangement of the erB-2 protooncogene in primary human stomach carcinomas. *Cancer Res.*, **49,** 6605–6609.

POWELL SM, ZILZ N, BEAZER-BARCLAY Y, BRYAN TM, HAMILTON SR, THIBODEAU SN, VOGELSTEIN B AND KINZLER KW. (1992). APC mutations occur early during colorectal tumorigenesis. *Nature*, **359,** 235–237.

POWELL SM, PAPADOPOULOS N, KINZLER KW, SMOLINSKI KN AND MELTZER SJ. (1994). APC gene mutations in the mutation cluster region are rare in esophageal cancers. *Gastroenterology*, **107,** 1759–1763.

RENAULT B, VAN DEN BROEK M, FODDE R, WIJNEN J, PELLEGATA NS, AMADORI D, MEERA KHAN P AND RANZANI GN. (1993). Base transitions are the most frequent genetic changes at P53 in gastric cancer. *Cancer Res.*, **53,** 2614–2617.

SANO T, TSUJINO T, YOSHIDA K, NAKAYAMA H, HARUMA K, ITO H, NAKAMURA Y, KAJIYAMA G AND TAHARA E. (1991). Frequent loss of heterozygosity on chromosomes 1q, 5q, and 17p in human gastric carcinomas. *Cancer Res.*, **51,** 2926–2931.

SHEFFIELD VC, BECK JS, KWITEK AE, SANDSTROM DW AND STONE EM. (1993). The sensitivity of single-strand conformation polymorphism analysis for the detection of single base substitutions. *Genomics* **16,** 325–332.

SPIRIO L, JOSLYN G, NELSON L, LEPPERT M AND WHITE R. (1991). A CA repeat 30–70 KB downstream from the adenomatous polyposis coli (APC) gene. *Nucleic Acids Res.*, **19,** 6348.

TAMURA G, MAESAWA C, SUZUKI Y, OGASAWARA S, TERASHIMA M, SAITO K AND SATODATE R. (1993). Primary gastric carcinoma cells frequently lose heterozygosity at the APC and MCC genetic loci. *Jpn J. Cancer Res.*, **84,** 1015–1018.

TAMURA G, MAESAWA C, SUZUKI Y, TAMADA H, SATOH M, OGASAWARA S, KASHIWABA M AND SATODATE R. (1994). Mutations of the APC gene occur during early stages of gastric adenoma development. *Cancer Res.*, **54,** 1149–1151.

UCHINO S, TSUDA H, NOGUCHI M, YOKOTA J, TERADA M, SAITO T, KOBAYASHI M, SUGIMURA T AND HIROHASHI S. (1992). Frequent loss of heterozygosity at the DCC locus in gastric cancer. *Cancer Res.*, **52,** 3099–3102.

UZAWA K, YOSHIDA H, SUZUKI H, TANZAWA H, SHIMAZAKI J, SEINO S AND SATO K. (1994). Abnormalities of the adenomatous polyposis coli gene in human oral squamous-cell carcinoma. *Int. J. Cancer*, **58,** 814–817.

VAN DER LUIJT R, MEERA KHAN P, VASEN H, VAN LEEUWEN C, TOPS C, ROEST P, DEN DUNNEN J AND FODDE R. (1994). Rapid detection of translation-terminating mutations at the adenomatous polyposis coli (APC) gene by direct protein truncation test. *Genomics*, **20,** 1–4.